# Supplementary material for: Are carbon emissions trading and green financial instruments synergistic? -Comprehensive quantitative research based on content analysis
Source: PLoS One. 2024 Mar 7;19(3):e0298601. doi: 10.1371/journal.pone.0298601 (PMC10919601; doi:10.1371/journal.pone.0298601)
Supplement: S1 Data — (ZIP) [file pone.0298601.s001.zip › Data/地级市Z_F工作报告.zip!/╔╜╬≈/┬└┴║╩╨/└┤╫╘╣1⁄2╓┌║┼í╛┬φ┐╦╩2╛▌═°í┐_2008.docx]

现在，我代表市人民政府向大会作工作报告，请予审议，并请各位政协委员和列席人员提出意见。

一、2007年工作回顾

2007年是我市全面落实科学发展观，加快构建和谐吕梁的重要之年。一年来，在中共吕梁市委的正确领导和市人大、市政协的监督支持下，我们坚持以科学发展观为统领，突出发展现代农业、深化结构调整、扩大对外开放、加强基础设施建设、推进社会事业发展、构建和谐社会等战略重点，深入实施“双百双千”和“环保攻坚”两大战役，戮力同心，奋发进取，取得了经济社会又好又快发展的重大胜利。

全市地区生产总值达到500.1亿元，增长16%，增速位居全省第二；人均地区生产总值达到14028元，比上年净增2575元。全社会固定资产投资完成301.1亿元，增长48.4%。工业增加值达到310亿元，增长18%。社会消费品零售总额达到125.5亿元，增长24%。外贸进出口总额达到6.75亿美元，增长40.8%。财政总收入达到105.1亿元，增长36.3%，总量跃居全省第四；其中一般预算收入达到38.3亿元，增长42.7%。城镇居民可支配收入达到10736元，增长16.3%；农民人均纯收入达到2778元，增长12.8%。居民消费价格总水平涨幅为5.1%。万元生产总值综合能耗下降7.0%，万元生产总值SO2、COD排放量分别下降20.2%、17.2%。城镇新增就业岗位3.96万个，城镇登记失业率为3%。在此，我郑重向大会报告，市一届人大五次会议提出的各项目标任务全面完成，市政府向全市人民承诺的“十件实事”全部兑现！

一年来，我们扑下身子，真抓实干，主要做了以下几方面工作并取得了显著成绩：

(一)经济结构调整继续深化，市域经济增长质量显著提高。通过工业主导、园区集聚、项目推动和循环经济牵动“四大措施”，着力推进工业新型化，工业经济实现了速度与质量的全面提升。全市新建和成长壮大的规模以上工业企业117户，规模以上工业增加值占地区生产总值的比重提高到50.2%。工业园区形成的总产值、增加值、利税在工业经济总量中的比重上升到“三分天下有其一”的水平。民营企业提供的就业岗位增加到44.12万人，民营经济增加值占地区生产总值的比重达到70%。有13户重点企业纳入了上市规划，各项前期工作进展顺利。建成省级循环经济试点工业园区3个、省级资源综合利用示范企业2户。原煤洗选率达到65%，焦炉煤气回收利用率达到62%。高度重视“三农”工作，新增财力的70%投向了“三农”领域，农业产业化水平进一步提高。农业新技术、新品种推广力度加大，大秋作物优种覆盖率达到85%以上，其中玉米、高粱优种覆盖率达到100%。吕梁山东西两麓旱垣区优质谷子基地、沿黄丘陵区名优豆类基地、南北冷凉区脱毒马铃薯基地初具规模，小杂粮基地面积达到308万亩，位居全省第一。年产值亿元以上、5000万元以上的农业产业化龙头企业分别增加到3个、5个。累计完成引导性培训15.36万人，新转移农村劳动力6.12万人，劳务创收50.3亿元，农民人均工资性收入达到1920元，同比增收405元。列入规划的整村推进和扶贫移民工程顺利实施并全部完成。新农村建设“十大模式”全面推广，80%以上的试点村和重点推进村初步形成了各具特色的产业。捆绑涉农项目、整合支农资金的做法得到了温家宝总理的肯定，机关企业结对帮扶新农村建设的经验在全省推广。第一产业增加值增速达到10.3%，增速近五年来最快。加快服务业发展提上了重要工作日程，一系列政策和工作措施显现出初步成效。

(二)环保攻坚力度空前加大，生态环境开始改善。借助“区域限批”东风，全面落实“环保攻坚年”各项措施，以壮士断腕的决心亮剑治污，一大批违规建设企业和落后产能被关停取缔，一大批违法排污企业得到有效治理。全年共削减SO25.88万吨、COD1.49万吨。空气质量明显改善，吕梁市区空气质量二级以上天数达到322天，由全省倒数第一跃升为正数第一。节能降耗工作全面铺开，焦化、钢铁、水泥三大行业累计淘汰落后产能1969万吨，节能181.7万吨标准煤。“造林绿化六大工程”和“三大经济林覆盖工程”均超额完成了任务。一幅山清水秀、空气清新、环境优美的“生态吕梁”画卷正在徐徐展开。

(三)对外开放和“双百双千”项目建设纵深推进，发展动力持续增强。在“珠洽会”、“煤博会”、“中博会”等重大招商活动中，共有43个项目对接成功，其中25个项目已开工。在提升产业项目招商水平的同时，积极引导市外资金投向基础设施和城乡公用事业，招商引资工作呈现出由“引资”变“选资”的历史性变化。备受全市人民关注的“双百双千”项目大会战，在立项、用地、环评、开工、筹资等五个关键环节上取得了重大突破，282个项目已经开工259个，开工率达到91.8%，其中75个项目全部完工，54个项目部分完工。这些项目的建设掀起了我市新一轮投资高潮，大大加快了传统产业优化升级步伐，为市域经济又好又快发展增添了强大的动力。

(四)基础设施建设捷报频传，特色城镇化发展步伐坚实。一批事关吕梁发展全局的路、水、电等重大基础设施建设项目完成改造或建成投运。太军高速公路全线贯通，沿黄旅游公路黑峪口至军渡段顺利通车，汾平、太佳两条高速公路正式奠基，县乡、乡村公路建设成果丰硕，公路密度比上年提高了5.7公里/百平方公里，最后16个偏远行政村结束了不通公路的历史。太中银、岢瓦、孝柳等铁路建设与改造工程进展顺利，吕梁民航机场即将立项开工。横泉水库枢纽工程喜获国家水利工程建设最高奖——“大禹奖”、一期供水工程即将竣工，柏叶口水库上半年有望立项开工，引文入川供水工程全面竣工，“6+1”水利工程全部实现了预期目标。柳电二期工程投入运营，吕梁500KV输变电工程进展顺利，一批220KV、110KV输变电工程相继建成，电力供应能力和电网运行水平正在发生历史性跨越。吕梁市区东川河蓄水及景观提升工程圆满完成，城区绿化亮化成效显著，凤山、滨河夜景广受赞誉，集中供热二期工程如期供热，垃圾处理厂和污水处理厂竣工投运。深入开展提升市民素质与创建卫生城市活动，城市管理能力和市民共建意识明显增强，顺利实现了省级卫生城市连创目标。各县(市)大力实施特色城镇化发展战略，县城和重点城镇建设与管理迈出了坚实步伐。

(五)和谐社会建设成效明显，“以人为本”执政理念深入人心。以“两免一补”为主的教育资助政策落实到位，农村中小学现代远程教育工程顺利实施，“双百”示范校建设步伐加快，普通高中办学条件继续改善，职业教育、成人教育稳步发展。市区教育基础设施建设取得实质性突破，吕梁实验中学高、初中实现分离办学，吕梁高级中学一期工程等一批新建、扩建工程如期竣工。公共卫生体系进一步完善，县、乡、村三级医疗卫生机构达标率达到58.7%。新型农村合作医疗试点工作深入推进，参合率达到90%以上，城市社区卫生服务迈出新步伐。科技创新能力不断提高，建成省级科技示范园区1个、市级示范园区7个，高新技术增加值占地区生产总值的比重提高到3.1%，每10万人专利申请数增幅居全省第一。“体育设施建设年”成效显著，全民健身运动广泛开展，成功承办了全省第六届农民运动会等大型赛事。基层文化建设进一步加强，民间文化艺术加快发展，广播电视和邮政通讯事业长足发展。社会保险扩面工作取得新进展，养老、医疗、失业、工伤、生育五项社会保险参保人数同比增加66.32万人。全年各类安全生产事故起数同比减少357起，死亡人数减少60人，煤矿事故死亡人数下降73%。国防和人民武装工作跨入全省先进行列。人口与计划生育工作第五次荣膺全省综合先进市称号。

(六)民主法制建设全面提升，发展环境进一步优化。主动接受人大法律监督、工作监督和政协民主监督，自觉坚持向人大报告工作，积极支持政协参政议政，认真听取并办理人大代表的建议、意见和政协委员的提案、建议。主动加强与各民主党派、工商联、无党派人士、各人民团体的联系，积极支持群团组织开展工作。不断加强普法教育，高度重视政府法制建设，严格落实行政执法责任制，稳步推进行政审批制度改革。全面落实政府系统党风廉政建设责任制，切实加强行政监察和审计监督。深入开展“作风建设年、狠抓落实年”活动，行政效能进一步提高。继续加强信访工作，认真落实市长接待日制度。严厉打击各类刑事犯罪，加强社会治安综合治理，初步实现了“发案少、秩序好、群众满意”的目标。重拳出击开展了打击非法采矿“百日铲除”行动，私挖滥采蔓延势头得到有效遏制。全力实施“治超载、保畅通”百日大会战，公路交通运输秩序明显好转。

各位代表，这些成绩的取得，是省委、省政府正确领导的结果，是全市人民同舟共济、奋力拼搏的结果，是社会各界大力支持、共同奋斗的结果。在此，我代表市人民政府向全市人民，向驻吕解放军指战员、武警官兵和中央、省直属单位，向所有关心、支持吕梁发展的各界朋友，表示衷心的感谢和崇高的敬意！

二、2008年经济社会发展的目标任务

方今吕梁，正处于转型跨越崛起的新的历史起点上，有着众多发展机遇和有利条件。一是世界经济对能源的依存度越来越大，国内市场对基础能源和钢铁、化工等产品的需求继续高涨，我市主导产业的市场形势仍然看好。二是国家深入推进中部崛起战略，继续加大对革命老区、贫困地区的支持力度，我省被确定为全国唯一的煤炭工业可持续发展政策措施试点省份，这为我们加快发展提供了前所未有的政策机遇。三是我市作为全省重要的能源化工和原材料工业基地，经过近几年的努力，已经积累了一定的产业基础和发展经验，大规模开发和承接东部产业转移的条件日趋成熟，加快发展的内动力十分强劲。当然，我们也要清醒地认识到，在发展过程中依然面临着许多困难和问题。一是国家实施稳健的财政政策和从紧的货币政策，不断加大建设用地供给、环境容量控制和节能减排考核等调控力度，对项目建设和企业经营形成了不同程度的影响。二是制约我市科学发展的县域、城乡、产业结构、经济社会“四大失衡”和交通、水资源、生态“三大瓶颈”问题仍未根本解决，煤矿安全生产压力依然很大。三是城乡居民收入与全国、全省相比仍有较大差距，人民群众对物价较快上涨的承受能力有限，消费拉动发展的能力还较为弱小。加之，我们在思想观念、体制机制和工作作风等方面还存在一些与转型发展、跨越发展不相适应的问题。为此，我们既要强化机遇意识，更要树立忧患意识，始终保持清醒的头脑，进一步解放思想，把握全局，科学谋划，趋利避害，扬长避短，在新的一年里继续保持经济社会又好又快发展的良好势头。

2008年是全面落实“十一五”规划、承前启后的关键之年。按照市委二届四次全会的部署，政府工作的总体思路是：以党的十七大精神为指针，深入贯彻落实科学发展观，不断引深“三大工程”建设，合力攻坚“双百双千”项目，继续坚持“开放引进、开发拉长、多元做大、科学发展”的经济结构调整思路，大力推进节能降耗和环境保护，加快新型工业化、特色城镇化、农业现代化进程，深化改革，扩大开放，改善民生，统筹城乡，努力开创经济社会又好又快发展的新局面。

按照这一总体思路，今年我市经济社会发展的主要预期目标是：

地区生产总值达到625亿元以上，人均地区生产总值达到18120元；全社会固定资产投资总额达到375亿元以上；财政总收入达到130亿元以上；工业增加值达到370亿元以上；社会消费品零售总额达到140亿元以上；外贸进出口总额达到7.5亿美元。

以上为指导性指标，我们还将着眼于优化结构、提高效益、降低能耗、保护环境，确保完成下列约束性指标：万元地区生产总值综合能耗下降4.5%；SO2排放量控制在8.7万吨以下，COD排放量控制在3.29万吨以下，吕梁市区空气质量二级以上天数力争比上年再有增加；居民消费价格总水平涨幅控制在4.7%左右；农民人均纯收入力争突破3100元；城镇居民人均可支配收入达到12000元；城镇新增就业岗位3.3万个，转移农村剩余劳动力5万人；城镇登记失业率控制在3%以内；新型农村合作医疗覆盖率达到100%，财政补助水平进一步提高；农村再解决13.7万人口的温饱问题；城市义务教育阶段免费教育全面实施；人口自然增长率控制在6.08‰以内。

根据上述总体思路和预期目标，今年将重点做好以下八个方面的工作：

(一)推进经济结构调整，加快转变发展方式

全力攻坚“双百双千”项目。按照“签约项目抓落实、未批项目抓审批、已批项目抓开工、开工项目抓进度、在建项目抓质量、建成项目抓投产”的思路，继续采取领导包项目、全程服务、跟踪监督等措施，逐一解决项目设计调整、手续报批、资金筹措、投产达效等环节的具体问题，继续加快各类项目的推进速度，力求尽快形成新的经济增长点。全力抓好兴县资源型循环经济综合开发示范基地和太钢岚县项目区等重点项目建设，努力使这些项目早日成为引领吕梁工业经济跨越式发展的“旗舰”。与此同时，继续把园区集聚作为优化产业结构的主要载体，紧紧扭住基础设施、骨干项目等关键环节，创新建设模式，提高管理水平，全力推动主导产业集群化发展。

着力提升工业经济水平。原煤行业，按照“生态修复、环境治理、资源整合、安全生产、转产转型”的要求，大力实施科技兴煤战略，重点在抓整合、深加工、保安全等方面下功夫。鼓励支持重点企业通过联合、兼并、重组等形式建设一批大型现代化矿井，推动煤炭行业规模化、集团化、洁净化、可持续发展。继续加大矿井技术改造力度，现有生产能力在30万吨以上的矿井，力争年内全部实现机械化开采，已经达标的矿井要向“两型三化”的更高目标迈进。焦化行业，严格执行国家新的行业和技术标准，尽快完成“双百双千”项目中不达要求项目的设计调整，争取早日立项。按照“控制总量、关小上大、提升档次”的要求，等量或减量置换现有污染严重、效益低下的落后产能，提高资源利用效率，减少污染排放。冶炼行业，按照新上项目高炉容积必须达到1000m3以上的要求，加快已完成置换审批手续的项目建设进度；按计划、分步骤关停落后产能，年内全部取缔200m3以下的炼铁高炉。采取产能置换方式，鼓励支持精深加工，延伸产品链条，提高产业竞争力。化工行业，坚持“拉链”与“补链”并重，大力推进以焦化副产品加工为主的煤化工业，积极支持焦油深加工、焦炉煤气制甲醇、合成氨、二甲醚、联醇等项目建设，抢占新型化工业的“制高点”，形成传统工业新型化的亮点。建材行业，大力调整产品结构，鼓励新型墙体材料生产，积极发展高标号水泥、特种水泥和轻质高温耐火材料，加快推进优质浮法玻璃、煤矸石制砖、粉煤灰建材制品等综合利用项目建设。高新技术产业，以发展装备制造、铝镁深加工为切入点，挂靠省内外大企业，积极引进新型适用技术，支持以企业为主体的技术创新，大力促进高新技术产业化发展。加快多晶硅工业化的进程，环磷酸酰酐尽快形成规模并占领市场，针状焦加快做大。

大力发展服务业。制定服务业发展规划，放宽准入门槛，放大投资领域，优化发展环境，加大政府引导性投入，以重点项目建设为着力点，确保全市重点支持的120个服务业项目半数以上年内开工，以此推动服务业加快发展速度，提升发展水平，力争服务业占地区生产总值的比重提高1个百分点。充分发挥离石区的龙头作用和东部四县(市)以及柳林、中阳等县的地缘优势和产业优势，加快培育和优先发展物流配送、金融保险、科技信息、中介会展等面向生产的服务业，促进服务业与能源化工和原材料工业有机融合、互动发展。坚持政策扶持和招商引资并重，力争在餐饮住宿、社区服务、娱乐休闲等面向民生的服务业方面取得新突破，年内引进1—2户国内知名商贸流通企业。积极发展面向农村的服务业，不断繁荣农村经济，提高农民生活水平。加快制定旅游业发展规划，完善激励政策和管理制度，扶持重点企业，规范行业秩序，挖掘特色文化内涵，引入战略合作伙伴，全力打造全省乃至全国具有影响力的旅游品牌，精心推出依托太原、对接晋中的精品旅游线路，尽快把资源优势转变为产业优势、经济优势，实现我市旅游产业的重大突破。

各位代表，产业结构畸重、增长方式粗放，是我市经济发展中最突出的问题。解决这一问题，非一朝一夕之功。只要我们自觉坚持科学发展观，坚定不移地落实经济结构调整这一打基础、利长远的战略措施，创新思路、奋力攻坚，吕梁产业转型、经济跨越、整体崛起的宏伟蓝图一定能够变成现实！

（二）积极发展现代农业，大力推进社会主义新农村建设

继续加大扶贫攻坚力度。以国家扶贫开发重点县为主战场，坚持“以工补农、以财扶农、以城带乡”的开放式扶贫思路，深入贯彻以产业扶贫为主的开发式扶贫方针，以帮助绝对贫困人口基本解决温饱和低收入人口稳定脱贫为目标，动员社会力量参与扶贫开发事业，重点实施“整村推进”、“移民搬迁”、“雨露计划”和“产业化扶贫”等四项扶贫工程，不断提高扶贫标准，提升扶贫开发水平。高度重视动物疫病防治，充分发挥气象技术手段的防灾减灾作用，扎实开展生产自救，认真解决农民群众因灾返贫的问题。

积极发展现代农业。全面贯彻工业反哺农业、城市支持农村和多予少取放活的方针，认真落实中央“三个高于”的要求，进一步加大“三农”投入力度，将基础设施建设和社会事业发展的重点转向农村，在小型农田水利建设、耕地综合生产能力建设、农村生态建设、农业科技创新与推广、新型农民培训、农村信息化建设等方面取得实质性成效，市本级用于“三农”的财政支出比上年增长32.8%。按照“5485”总体目标，继续发展特色高效农业，提高农业规模化、集约化、产业化水平。大力建设一批现代农业园区和特色种养业生产基地，尽快形成若干个具有品牌效应、产业集聚效应和典型示范效应的优势产业带和产业区，夯实农业增效、农民增收的产业基础。采用贷款贴息等措施，做大做强产业化龙头企业，大力发展农民专业合作社，推进农业产业化进程。积极探索“公司+基地+农户+保险”的经营模式，健全风险共担、利益共享的农业产业化机制。大力实施“菜篮子”工程，扶持发展城郊蔬菜基地，保障城乡蔬菜市场供给。进一步加强无公害、绿色、有机农产品和基地的认证工作，引导和支持特色农副产品进入城市消费市场，不断提高劳务经济的组织化程度，打响吕梁特色农业品牌和劳务品牌。

大力推进新农村建设。坚持“政府引导、群众为主、因地制宜、量力而行、循序渐进、务求实效”的原则，全力支持117个试点村、226个重点推进村以“一村一品”为重点的产业开发，不断增强其产业带动能力。深入推进“治五乱、达五化”农村环境整治工作，积极改善农村生活环境。进一步完善新农村建设模式，继续抓好企业帮扶、单位帮建、项目捆绑、资金整合等政策措施的落实，不断完善多元投资、社会共建的推进机制，让更多群众享受新农村建设的成果。

各位代表，“三农”问题直接涉及到我市近70%人口的利益，始终是我们工作的重中之重。“三农”问题的实质是农民增收，重点是推进社会主义新农村建设，抓手是实施农业产业化、推进农业现代化。我们将继续坚定不移地致力于“三农”工作，使农业发展更快、农民增收更多、农村更加繁荣稳定。

(三)大力推进节能减排，积极建设生态文明

全面加强节能降耗工作。以工业节能和淘汰落后产能为主要抓手，扎实推进各项工作。建设一批大项目、好项目，改造提升煤、焦、冶金等传统产业，加快发展煤化工、铝镁、新型建材等新兴产业，并对重点节能企业给予差别电价、水价等政策倾斜；发展一批循环经济典型县和循环经济示范区，努力形成若干个循环经济产业带、企业群，着力推进矿产资源高效利用、固体废物综合利用、再生资源循环利用以及水资源再利用，逐步实现生产集聚化、集约化；引进推广一批节能降耗关键技术和共性技术，推动以企业为主体、产学研相结合的节能技术创新与成果转化体系建设；淘汰一批能耗高、污染重、效益低的落后产能，按照分县(市、区)、分年度强制淘汰落后产能的具体方案，责任到企业到部门，倒排时间，限期完成，最大限度地为大项目、好项目腾出容量。同时，积极推进建筑、交通、商务、行政等领域的节能工作。市政府将全面建立和完善节能降耗统计体系、监测体系和考核体系，严格执行主要耗能产品能耗限额，严格执行节能问责制和“一票否决”制，强化监督管理和责任考核，以坚定不移、务求必胜的决心和干劲把节能降耗的各项目标任务不折不扣地完成好。

启动第二个环保攻坚年。继续发扬敢打硬仗、一往无前的精神，全面完成环保攻坚第二战役尾欠的各项治理任务，奋力打好环保攻坚第三战役。彻底取缔市区剩余443台燃煤供暖锅炉，新增600万平方米的集中供热面积，加大施工扬尘和道路扬尘整治力度，努力让市区居民摆脱烟尘、粉尘的侵害。进一步加大干线公路、主要河流沿岸、饮用水源保护区以及城市周边排污企业的整治监管力度，督促各类企业建设并运行污染源在线监测监控系统，实时监控环境敏感区重点工业污染源，对排放不达标的企业坚决依法关停。下大力气查处土小污染企业死灰复燃的问题，依法严惩快处那些利欲熏心、铤而走险的不法分子。加大水污染防治力度，建成文峪河、三川河等重点河流沿线的5个城市（县城）污水处理厂，并确保正常运行。积极推进环境科技创新，强化各类企业社会责任，唤起全民环保意识，运用科技手段、依靠各方力量，不断提升环境保护工作水平，早日再现吕梁碧水蓝天。

深入推进水土保持和造林绿化。加强以淤地坝建设为重点的水土保持工作，抓好沿黄乡镇水土治理与保护，扎实开展重点流域治理，年内新建淤地坝100座，完成水保初治面积95万亩。认真落实退耕还林还草政策，加大封山禁牧力度，继续抓好退耕还林、天然林保护、三北防护林建设等国家重点工程和省级重点工程，扎实推进“三大经济林覆盖工程”。积极争取上级支持，巩固和扩大造林绿化“六大工程”的成果，新增造林绿化面积80万亩。深入推广“一企绿化一山”、“一厂建设一带”的造林模式，调动各方面的力量建设山川秀美的生态环境。

加强地貌治理与资源保护。坚持“谁开发谁保护，谁破坏谁治理”原则，做到边采矿、边整治、边复垦，严肃查处违法违规行为。以居民住宅、学校、医院、道路、土地平整为治理重点，大力整治危及人民群众生命财产安全的地质灾害。建立采煤沉陷区治理长效机制，把地貌治理与保护工作纳入资源开发和生产规划当中，坚决纠正先破坏、后治理，先污染、后治理的“末端治理”模式。制定和完善打击非法违法采矿的制度规定，尽快形成规范采矿秩序的长效机制。继续引深打击非法违法采矿“百日铲除行动”，深度挖掘纵容包庇不法分子的“保护伞”，坚决铲除公职人员中贪赃枉法、顶风违纪的“害群之马”！

各位代表，今年是完成“十一五”节能减排约束性指标的关键之年。党中央、国务院给各级政府下了“军令”，广大人民群众对建设生态文明有着热切期盼。我们将运用综合手段，采取切实措施，坚决完成好这些事关吕梁对外形象和投资环境，事关全市人民生活水平和生存质量的攻坚任务，向省委、省政府与全市人民交上一份满意的答卷！

(四)加强基础设施建设，推动城乡统筹发展

全面加快交通建设步伐。大力推进太佳、汾平高速公路建设，全面完成307国道以及桃临线、孝石线、汾孝大道、沿黄干线吕梁段、离(石)碛(口)旅游路、沿黄扶贫旅游公路二期、古交至岚县二级公路等重点项目的改造与建设任务，完成交城、文水、汾阳、交口、临县、柳林等绕城公路工程建设任务。开工340省道汾阳至离石段一级路改造、离石环城一级路建设等工程。积极筹划兴县至交口高速公路的前期工作。加快太中银铁路、岢瓦铁路建设进度，力争年内开工太中银吕临支线、太古岚铁路、介西支线的建设与改造工程，配合省政府完成好途经兴县、临县、柳林、石楼等县的铁路出海大通道筹备工作。开工建设吕梁民航机场。通过这些重点工程的建设，尽快形成一个公路、铁路、民航互为支撑的立体交通体系。

继续加大水利建设力度。按照“合理开发、高效利用、全面节约、有效保护”的总体要求，创新投融资机制，深入实施兴水战略。横泉水库供水工程上半年投入运行。加快千年水库、坪底水库建设步伐，争取开工建设柏叶口水库、魏家沟水库、阳湾子水库、高红水库等一批中小型水库，全面完成病险水库除险加固任务。积极推进沿黄工业取水、农村安全饮水等重点工程。

进一步加快电源电网建设。千方百计争取国家和省的支持，加快我市综合利用发电项目建设，年内争取开工建设柳电三期、兴县和石楼两座2×60万KW、离石2×30万KW热电联产以及交城、岚县两座2×30万KW煤矸石电厂等重大电源项目。注重电力外输能力建设，加快电网建设和改造步伐，确保吕梁500KV输变电工程和岚县、兴县、交口、孝义等220KV输变电工程如期建成，确保广兴220KV增容工程上半年竣工投运，确保列入规划的13个110KV输变电工程和4个110KV变电站增容改造工程建成投运，加快柳林、离石220KV输变电工程建设。

提升中心城市品位。以建设“宜居城市”为目标，调整城市总体规划，修订控制性详细规划和各类专业规划，严格各类规划管理，依法查处乱修乱建，稳步推进城中村改造。改革公用事业经营体制，引导社会资本参与市政公用设施建设。继续实施“三山绿化”工程，重点抓好北川河综合整治、滨河北路东延以及步行街、八一街、交通路、高速公路西出口等街路的改造提升，加快市区边山边沟排洪、市区污水处理厂二期、东川桥、石州桥拓宽等市区工程的建设进度，不断完善城市功能。科学布局并积极建设市区停车场地，解决市区停车难的问题。加快东、西过境公路选线设计工作，争取早日开工建设。继续开展提升市民素质和创建卫生城市活动，进一步提升城市品位。

培育现代文明城镇。继续加大各县(市)城区的建设和管理力度，加快城镇交通、商贸、通信、水网、绿化、科教文卫配套设施建设，完善县城、中心乡(镇)功能，增强城镇综合承载力。依托工业园区和重大项目建设，突出产业优势和文化特色，加快建设一批工业重镇、商贸集镇、旅游大镇、文化名镇，繁荣城乡经济，促进统筹发展。

各位代表，城乡经济社会发展一体化，是党的十七大提出的新要求。我们将始终坚持把统筹城乡发展作为政府工作的重要任务，在不断突破瓶颈制约，改变我市交通、水利、电力条件的基础上，进一步完善城镇功能，提升中心城市辐射力、带动力，壮大一批中心集镇，努力走出一条资源型城市特色城镇化发展的新路来！

(五)继续深化改革，全方位对外开放

深化各项综合改革。进一步加快国有企业改革步伐，支持非公有制经济和混合所有制经济发展。规范国有资产监督管理，确保国有资产保值增值。改善政府投资管理模式，提高财政支出效益。深化公共事业运营体制改革，鼓励民间资本投资公共事业。推进农村综合改革，强化农村财务管理，稳定和完善农村土地承包关系，探索土地承包经营权流转的市场机制。加快农村金融改革，支持农村信用社持续发展，抓好村级发展互助基金试点、小额贷款试点以及扶贫资金整合使用推广工作。

全面推进金融创新。尽快修订完善全市金融发展规划，大力支持地方性商业银行建设，积极引进浦发、民生等股份制商业银行来吕梁设立分支机构。大力拓展资本市场，重点在企业上市、中小企业捆绑发行债券、建立投资基金和筹建碳排放交易所等四项新工作上取得突破。积极支持楼东俊安、山西联盛、大土河集团、东辉集团等13户重点企业上市前的各项工作，力争年内有2—3户企业上市融资。

拓展对外开放领域。实施“向东”开放战略，重点在环渤海经济圈尤其是首都地区求突破，扩大长三角、珠三角及港澳地区的招商引资规模；实施“向中”开放战略，借助中部崛起的政策机遇，努力在加强与中部六省的区域合作方面取得新成效；实施“向西”开放战略，大力推动各类生产要素与西部各省(区)的深度对接。落实“三向”开放战略，将重点从以下三方面着手：一是深入推进与北京朝阳区、北京理工大学的友好合作，办好山西吕梁(北京朝阳)招商引资项目推介暨特色农副产品展示会，力求在利用首都地区的资金、技术、人才等资源方面取得重大突破。二是继续抓好晋沪吕梁煤化工园区建设的各项工作，力争规划建设的项目尽快落地生根、达产达效、形成规模。三是有针对性地参加国内各类大型招商引资和招才引智活动，在集聚各类资本的同时广揽天下英才，在扩充经济总量的同时提升城市软实力。

各位代表，改革开放是新时期最鲜明的特点，也是经济社会发展的不竭动力。对于吕梁而言，改革的深度和开放的广度，都有很大的拓展空间。为此，我们将进一步解放思想，开拓创新，不为任何困难所惧，不被任何干扰所惑，用改革的思维解决转型中的问题，借开放的力量弥补发展中的不足，使吕梁经济社会发展全面焕发出勃勃生机和持久活力！

(六)强化社会公共事业发展，努力建设和谐社会

优先发展教育事业。全力推进义务教育标准化建设，全面落实义务教育经费保障机制。加大农村中小学布局调整和寄宿制学校建设力度，加快城区学校改扩建步伐，进一步缓解入学难和班容量大的问题。提升教师队伍素质，合理配置城乡师资力量，促进义务教育均衡发展。认真解决农民工子女和农村留守儿童的教育问题。积极建设与主导产业发展相协调、与劳动力转移需要相适应的职业教育体系,充分发挥职业教育对提升城乡劳动力素质的作用。发展吕梁高等教育，加快建设吕梁学院。

加快发展科技事业。高度重视科技普及工作。加快推进以企业为主体、市场为导向、产学研相结合的区域技术创新体系建设，全面加强重点领域的自主创新和应用型技术推广，切实把科技成果转化为现实生产力。着眼于改造传统产业、培育新兴产业、发展高新技术产业，重点在引进、消化、吸收、再创新等方面取得突破，不断提高科技对经济增长的贡献率。年内争取每10万人专利申请数达到5项，新型工业化水平达到40%。

大力发展卫生事业。实现新型农村合作医疗制度全覆盖，进一步完善管理和监督机制，让260万参合农民真正享受实惠。继续改善农村医疗卫生条件，强化城乡卫生服务网络、应急救援体系和城市社区卫生服务体系建设，加强重大传染病预防控制。深入开展爱国卫生运动和食品卫生安全工作，着力提高广大群众的疫病预防水平。大力整治非法行医、开单提成、制售假劣药品等问题，保障全市人民放心就医、安全用药。

统筹发展文化体育等事业。编制文化产业发展规划，积极挖掘传统文化内涵，开发具有吕梁地方特色的文化产业，不断提升区域文化软实力。大力弘扬以爱国主义为核心的民族精神和以改革创新为核心的时代精神，丰富“吕梁精神”的内涵，鼓舞全市人民保持自强不息、昂扬向上的精神风貌。加强非物质文化遗产的申报与保护，严厉打击各种破坏盗窃文物行为。抓好农村广播电视“村村通”、城市电视数字化覆盖和知识产权保护等工作，不断繁荣广播电视、新闻出版等社会事业。积极启动文化扶贫工程。加快吕梁游泳馆、市体校综合教学大楼等重点体育基础设施建设步伐，积极开展全民健身活动和竞技体育运动，办好第二届全市运动会。继续抓好人口和计划生育工作，稳定低生育水平，提高人口素质。认真抓好国防教育和国防后备力量建设，积极发展人民防空事业，充分发挥民兵在新农村和“平安吕梁”建设中的作用，做好拥军优抚工作。大力发展测绘、气象、防震减灾、档案、史志、民族宗教和侨务等各项事业。

各位代表，社会事业发展是统筹发展的主要内容，也是我们必须高度关注并不断强化的薄弱环节。市政府将进一步调整财政支出结构，不断加大对社会事业的投入强度，努力为全市人民提供更多、更优质的公共产品和社会服务，确保社会事业与经济建设协调发展！

(七)着力改善民生，维护好广大群众特别是弱势群体的切身利益

继续扩大就业。坚持积极就业政策，鼓励支持劳动者自主创业和自谋职业。全面加强就业再就业服务体系建设，把充分吸纳劳动力作为政府扶持企业的重要条件。积极发展劳动密集型产业，推动多渠道就业，切实解决大中专毕业生、退伍军人、城镇“零就业”和农村“零务工”家庭以及“4050”人员的就业问题。

保障弱势群体基本生活。坚持广覆盖、保基本、多层次、可持续的方针，加快建立覆盖城乡的社会保障体系，千方百计解决困难群众的生产生活问题。提高农村困难群众的扶持比例，实现城乡救助比例均等化，扩大农村困难群众享受低保的范围，在应保尽保的同时，根据物价上涨水平，适时提高补贴标准。扎实做好社会保险扩面工作，积极启动城镇居民医疗保险。加强劳动监察和劳动用工管理，严肃惩处克扣、拖欠工程款和农民工工资问题，严厉打击非法用工、奴役民工行为。加快发展社会福利、残疾人保障和慈善救助事业，抓好法律援助工作，让弱势群体充分享受“公共财政”的阳光和社会主义制度的温暖。

扎实抓好安全生产。按照“安全第一，预防为主，综合治理”的方针，认真研究和落实治本之策。严格执行安全生产责任制，大力加强以煤矿、道路交通为重点的安全生产工作，认真抓好安全隐患排查整治，严厉打击超层越界开采、超能力超定员开采行为，坚决杜绝重特大事故发生。完善应急救援体系，提高应急处置能力，努力把事故和灾害损失降到最低限度。

各位代表，“民为邦本，本固邦宁”。促进社会和谐、保障民生安定始终是政府的首要责任。我们将坚持“以人为本”，崇尚公平正义，始终把实现好最广大人民的根本利益作为政府一切工作的出发点和落脚点，努力让全市人民共享吕梁改革发展的成果！

(八)推进政府体制和管理创新，加快建设管理服务型政府

坚持科学决策，提高服务水平。敏锐把握经济社会发展趋势，正确认识不断变化的市情、社情、民情，科学总结近年来改革发展的经验教训，不断深化对执政为民本质的认识，在解放思想中创新思路，把科学发展观自觉贯彻落实于政府行政行为的每一环节。创新行政管理体制，推进政企分开、政资分开、政事分开、政府与市场中介组织分开。健全政府职责体系，全面正确履行政府职能。积极推进政务公开，增强政府工作透明度和公众参与度，依法保障公民的知情权、参与权、表达权、监督权。积极推进电子政务建设，完善政务网络平台，落实政府信息公开承诺。建立和完善公众参与、专家论证和政府决策相结合的决策机制，提高科学决策的能力。开展绩效评估、绩效审计、行政问责“三位一体”的管理创新，切实解决行政效率低、成本高、失误和过错无人负责的问题。

加强社会管理，狠抓薄弱环节。着力增强政府解决各类社会问题的能力，积极维护社会公平，努力消除因分配不公、机会不均引发的不稳定因素。健全和完善基层社会管理体制，积极创建和谐社区、和谐矿山、和谐村庄。切实加强社会治安综合治理，继续推进“平安吕梁”创建活动。严厉打击各类刑事犯罪活动，加强基层派出所、司法所和基层法庭建设，提高维护稳定的保障能力。坚持信访接待制度，完善社会利益协调和矛盾纠纷排查调处机制，预防和化解社会不稳定因素。制定和完善公共突发事件应急预案，建立预警预报信息系统和应急处置平台，提高应急处置能力。积极探索“治超载、保畅通”长效机制，全面构建安全规范的道路运输秩序。

规范行政行为，推进依法行政。积极推进民主政治建设，认真执行人大及其常委会的各项决议、决定，主动向人大报告工作，自觉接受人大的法律监督、工作监督。密切同人民政协、各民主党派、工商联、无党派人士的联系，支持政协充分履行政治协商、民主监督、参政议政职能。认真办理人大代表、政协委员的议案、提案和建议，虚心听取社会各界的意见。支持人民法院、检察院依法履行职责，支持工会、共青团、妇联等群团组织依照法律和章程开展工作、参与社会管理。扩大基层民主，完善民主管理，保障人民合法权益。深入开展“五五”普法教育，加快推进依法治市进程。建立健全行政执法责任制、过错追究制、监督评议制等制度，保障行政执法部门和执法人员规范高效履行职责。坚持依法治税，加强税收征管，整顿和规范税收秩序。建立阳光财政，规范资金监督管理，提高财政资金的使用效益和安全性。

强化作风建设，提高执行力。把抓落实作为推动工作的着力点，切实加强行政效能建设，完善目标责任管理办法和激励机制，加大对政府部门履行职责的检查、监督与考核的力度，对重大部署、重要决策、重点项目、民生工程的落实和完成情况进行综合绩效考评，形成层层抓落实、事事要交帐的工作机制，决策了的事情雷厉风行、抓紧实施，部署了的工作一抓到底、抓出成效。大力弘扬求真务实的工作作风，精简会议和文件，深入调查研究，反对不求实效、只图虚名，不求有功、但求无过的形式主义、官僚主义。坚持为民用权、为民谋利，多做能让多数群众受益的实事，带着感情解决好老百姓最关心、最现实、最直接、最迫切的问题。

建设廉洁政府，提高公信力。按照“一岗双责”的要求，全面落实政府系统党风廉政建设责任制，建立健全教育、制度、监督并重的惩治和预防腐败体系。在坚决惩治腐败的同时，更加注重治本，更加注重预防，更加注重制度建设，努力拓展从源头上防治腐败的工作领域。严格执行廉洁从政各项规定，构建完善、有效的廉政监督机制和考核评价机制。强化行政监察和审计监督工作，严肃查处重点领域、重点人员的违纪违法案件，切实防止以权谋私，坚决维护群众利益。认真贯彻落实《公务员法》和《公务员处分条例》，严肃处理不作为、乱作为，玩忽职守，造成恶劣影响和严重后果的责任人。强化诚信意识，狠抓政府承诺事情的落实，取信于民，凝聚人心，调动和激发最广大人民群众的积极性、创造性。

各位代表，政府的权力由人民赋予，政府的使命就是为人民服务。加强政府自身建设，推进政府管理创新，归根结底就是要常怀爱民之心，勤思富民之策，笃行利民之举，以为人民办好事的好形象赢得人民群众的信任和支持！

现在，我将向大会郑重承诺，今年市政府在完成好上述工作任务的同时，还将重点围绕“民生”问题，为全市广大人民再办“十件实事”：一是新建、改建、扩建中小学校70所，为每县(市、区)1所新建的寄宿制学校配齐桌椅。二是县、乡、村三级医疗卫生机构基础设施70%达标，城市社区卫生服务机构建设完成80%以上。三是完成农村劳动力引导性培训10万人，转移5万人，新增城镇就业岗位3.45万个。四是新建经济适用住房15.5万平方米，解决2580户城市低收入家庭的住房困难；新建廉租住房4万平方米，让800户保障对象住上新房。五是在巩固成果的同时，再解决26万人的饮水安全问题。六是继续实施“万村千乡”市场工程，完善14个配送中心功能，提高配送比例，新建标准化农家店250个。七是新发展农村沼气用户3000户。八是再改造县乡公路500公里，完成村村通水泥路1000公里，年底行政村通水泥路率达到68%。九是再完成扶贫移民搬迁11000人。十是新建残疾人康复就业服务中心1所，为50个社区配备康复器材，为200户残疾人进行危房改造，为4000名贫困残疾人配备用品用具，对1000名贫困残疾人实施康复救助，对1000名特困残疾人家庭子女上学进行救助。

各位代表！时代赋予我们庄严使命，人民寄予我们热切期望。让我们高举中国特色社会主义伟大旗帜，深入贯彻党的十七大精神，认真落实科学发展观，在中共吕梁市委的坚强领导下，开拓进取，扎实工作，在建设经济繁荣、社会和谐、人民富裕、环境友好新吕梁的征程上，谱写更加绚丽的篇章!
